# Significance of upper airway influence among patients of vocal cord dysfunction for its diagnosis: Role of impulse oscillometry

**DOI:** 10.4103/0970-2113.45197

**Published:** 2009

**Authors:** H. S. Hira, Anshu Singh

**Affiliations:** *Pulmonary Medicine and Sleep Center, Department of Medicine, Maulana Azad Medical College and Lok Nayak Hospital, New Delhi - 110 002, India*

**Keywords:** Forced oscillation technique, flow volume loop, impulse oscillometry, pulmonary function tests, vocal cord dysfunction

## Abstract

**Background:**

To identify the patients of bronchial asthma (suspected or proven), not responding to optimal therapy, for the presence of vocal cord dysfunction (VCD) and to compare the diagnostic ability of flow volume (FV) loop and impulse oscillometry (IOS).

**Materials and Methods:**

Fifty one patients of suspected/proven bronchial asthma not responding to optimal therapy were included for the study. Each patient was subjected to both FV loop and IOS studies. Direct visualization of the vocal cords with flexible fiberoptic bronchoscope for the presence of inspiratory vocal cord adduction during quiet respiration, with speech, and while performing provocative maneuvers was carried out. All patients were subjected to simple pulmonary function tests and recording of FV loop. IOS was performed on each patient to look for the site of obstruction and upper airway influence. The observations of both FV loop and IO studies were compared.

**Results:**

Among 51 patients participated, 12 (23.53%) had bronchoscopical evidence of VCD and were labeled as VCD-positive group and rest 39 were designated VCD negative. No statistically significant difference in pulmonary function test (prereversibility) results between the VCD-positive and VCD-negative patients was found. Reversible airway obstruction was observed in 75% of the patients of VCD-positive group and 67.65% of the patients in the VCD-negative group. Only one patient in the VCD-positive and none in VCD-negative group had inspiratory limb flattening of FV loop. Upper airway influence was evident by IOS in 58.3% of patients in the VCD-positive group and in 15.4% of patients in the VCD-negative group. This difference was statistically significant (*P* **<** 0.005).

**Conclusion:**

VCD was a common finding in patients with symptoms suggestive of asthma and frequently coexists with asthma. IOS was found to be a useful screening test for VCD and was more sensitive than FV loop.

## INTRODUCTION

Vocal cord dysfunction (VCD) is characterized by paradoxical closure of the vocal cords with resultant airflow limitation at the level of the larynx.^1^ Adduction of true vocal cords usually occurs during inspiration in patients of VCD, but it may also occur during expiration.^2^,^3^ This adduction of the vocal cords during inspiration limits the airflow, and the patient complains of wheezing, stridor, shortness of breath, or dyspnea.^4^

The symptoms of VCD mimic a variety of upper and lower airway diseases.^5^ In various studies where a diagnosis of VCD was made, the initial diagnoses were asthma, exercise-induced bronchospasm, chronic obstructive pulmonary disease (COPD), anaphylaxis, and hoarseness.^1^,^3^,^4^ The symptoms, however, most often imitate the respiratory abnormalities found in asthma and patients are often treated for the same with unsatisfactory results.^1^ Retrospective studies have demonstrated that VCD can also coexist with asthma and response to the asthmatic medications may be inadequate or require higher drug doses.^1^

Making the diagnosis of VCD other than with direct visualization of the vocal cords can be difficult.^4^ Awareness along with a high index of suspicion is prerequisite for identifying this great mimicker of a disease. Pulmonary function tests may show a lack of airflow limitation between symptomatic episodes. VCD is suspected among patients with difficulty to treat asthma or who have no or suboptimal response to therapy.^4^,^6^ In the subgroup of patients with coexisting asthma and VCD, the spirometry may only reveal reversible airway obstruction characteristic of asthma.^1^

Flow volume (FV) loop in patients with VCD may demonstrate the truncation of the inspiratory limb, suggestive of variable extrathoracic obstruction.^5^ The sensitivity of FV loop is very low to diagnose VCD. The FV loop may not be very useful in patients of VCD with coexisting asthma; in such cases, expiratory flow limitation due to asthma will cause truncation of expiratory limb of FV loop as well.

The ‘gold standard’ for the diagnosis of VCD is the direct observation of the paradoxical inspiratory vocal cord closure while the patient is in the midst of an attack.^7^ The direct visualization may be carried out using a flexible bronchoscope or rhinolaryngoscope. The findings are inspiratory vocal cord closure with posterior ‘chinking’ (a small opening at the posterior aspect of the cords), although closure of the vocal cords can be seen during expiration as well.^1^,^8^

Impulse oscillometry (IOS) is based on the forced oscillation technique (FOT) which is a convenient method of determining the impedance of total respiratory system.^9^ The total respiratory impedance (*Z*_rs_) is partitioned into a ‘real’ (or resistance) *R*_rs_ and ‘imaginary’ (or reactance) *X*_rs_ component. The *R*_rs_ and *X*_rs_ are determined for several frequencies of oscillations. Various lower and upper airway disorders produce characteristic changes in *R*_rs_ and *X*_rs_ over the frequency spectrum used.^10^,^11^ The major problem of the observations of FOT is that it is affected by the shunt characteristics of the upper airway as oscillations applied at mouth level is ‘lost’ in movements of the cheeks and influences the values of *R*_rs_ and *X*_rs_.^10^,^11^ In the Mead's model of lung, this is termed as oropharyngeal compliance (*C*_m_). During the ongoing other project on IOS at our center, we realized that *C*_m_ may influence the observations. Therefore, we attempted to use this shunt as diagnostic tool to diagnose VCD and coined the term as upper airway influence.

## MATERIALS AND METHODS

The study was conducted in the division of Pulmonary Medicine of a tertiary referral and teaching institution. This consisted of 51 patients of suspected/proven bronchial asthma, not responding to optimal therapy. Those more than 12 years of age and either sex, attending the medical OPD, chest clinic, or emergency department were included. Academic review board of the institution approved the study protocol. Written and informed consent was sought from all participants.

Patients who either required high doses of oral steroids for the control of asthma symptoms or had an inconsistent response to bronchodilator therapy were taken for study. Subjects who had history suggestive of COPD, interstitial lung disease, chest wall deformities, and significant fibrosis or volume loss of lungs were excluded. Those with organic upper airway obstruction (e.g. that due to masses, polyps, and cysts) or with identifiable cause of VCD (brainstem compression, myasthenia gravis, neurological injuries, or other such causes) were also excluded.

All participants underwent a complete history and physical examination. They were subjected to pulmonary function tests with recording of forced vital capacity (FVC), forced expiratory volume in first second (FEV_1_), forced expiratory flow after exhalation of 50% and 75% of vital capacity (FEF50% and FEF75%, respectively), peak expiratory flow (PEF), and FV loop using an electronic spirometer (Master screen diffusion, JAEGER, Germany). For all these parameters, the actual/predicted ratio was computed. The criterion used for the diagnosis of airway obstruction was a FEV_1_/FVC ratio of less than 75%, and reversibility was carried out by using inhaled bronchodilators.

The flattening of the inspiratory limb of the FV loop was interpreted directly from the FV tracings by comparison of the mid-expiratory flow and mid-inspiratory flow at 50% of the FVC. A mid-inspiratory flow that was less than mid-expiratory flow at 50% of the FVC was taken as flattening of the inspiratory limb of the FV loop.

IOS study was performed on the Master screen diffusion, JAEGER, Germany. The output from IOS included tidal volume, resonance frequency, *Z*_rs_, *R*_rs_, and *X*_rs_ at frequencies of 5 Hz, 20 Hz, and 35 Hz, central (*R*_c_) and peripheral resistance (*R*_p_), *Z*_rs_ versus volume (*Z–V*) graph, resistance and reactance versus frequency spectra, and the visual interpretation graph (Lung Model) based on the seven element model of ‘lung of Mead’. The interpretation graph was the graphically expressed form of the interpretation of the impedance spectrum. In the present study, the appearance of oropharyngeal compliance (*C*_m_) on the visual interpretation of graph was considered as upper airway influence.

Each patient underwent bronchoscopy using a flexible fiberoptic bronchoscope. Topical 2% lignocaine was used to anesthetize the nares. The posterior pharynx was specifically not anesthetized to avoid medication of the vocal cords. The bronchoscope was directed to the posterior pharynx several centimeters above the glottis to prevent the stimulation of the vocal cords to induce their adduction. The motion of the vocal cords was documented with speech and any abnormal motion of the vocal cords was looked for with panting, sniffing, and deep breathing maneuvers each lasting for ten seconds. The observation of VCD was made in patients who showed adduction of vocal cords with one or more of these maneuvers during bronchoscopy.

For statistical analysis, patients were divided into two groups, VCD positive and VCD negative depending on the presence or absence of VCD, respectively, on bronchoscopy. The *t*-test/ Mann-Whitney test was used for assessing the difference between the two groups for the continuous variables, while Pearson chi square test was used for assessing the difference among discrete variables.

## RESULTS

Of 51 patients studied, 12 patients (23.53%) had evidence of VCD on bronchoscopy and were grouped as VCD positive. The remaining 39 patients were classified as the VCD-negative group. A comparison of the PFT, FV loop, and IOS observations of both groups was computed.

Seven (58.3%) of the patients were women, while rest were men. In the VCD-positive group the mean age was 32.92 ± 12.42 years (range 17–57 years). In the VCD-negative group, the mean age was 32.87 ± 10.928 years and 24 (61.5%) were females.

In the VCD-negative group, five patients could not perform the spirometry, while in the VCD-positive group all could do. Patients, who could perform, were included for the purpose of statistical analysis of spirometry parameters. The diagnosis of asthma was confirmed spirometrically in nine (75%) of the patients in the VCD-positive group and 26 (67.65%) of the patients in the VCD-negative group, as evidenced by significant reversibility of airway obstruction. There was no significant difference between the VCD-positive and VCD-negative groups in the spirometric parameters. Only one patient in the VCD-positive group and none among VCD-negative had inspiratory limb flattening of the FV loop.

**Figure 1:**
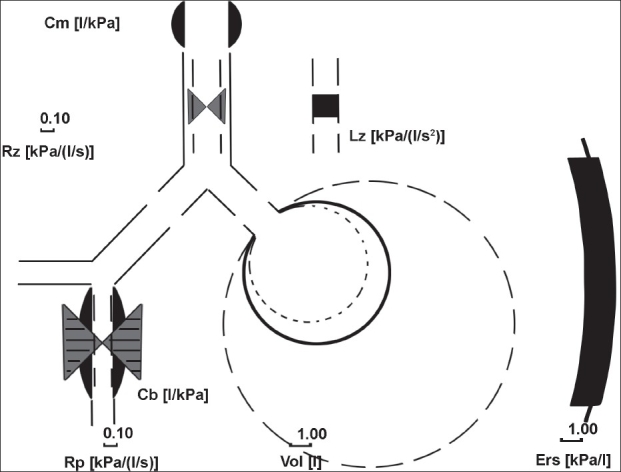
Interpretation graph (model of lung) of a patient showing oropharyngeal compliance C_m_, central component with central airway resistance R_c_, central inertance L_z_, bronchial compliance C_b_, peripheral component with peripheral airway resistance R_p_, lung compliance C_l_, and thoracic wall compliance C_w_. C_m_ (upper airway influence) shown is prominent

In the present study, upper airway influence [Figure 1] was evident on IOS in seven (58.3%) of the patients from the VCD-positive group and in six (15.4%) from the VCD-negative group. This difference was statistically significant (*P* < 0.005).

## DISCUSSION

VCD is increasingly being identified as a cause of recurrent episodes of cough and wheezing. Patients with VCD often receive a diagnosis of poorly controlled asthma with suboptimal response to therapy with β2-agonists and corticosteroids.^1^ Most of the patients with VCD were young females with a history of psychologic illness and/or childhood abuse.^1^

The majority of literature concerning VCD consists of small retrospective series and case reports. The only prospective study that evaluated VCD in a cohort of patients with complaints of exertional dyspnea reported 15% prevalence of VCD.^4^ The literature considered VCD to be relatively a rare condition that may mimic asthma or coexist with it. To authors' knowledge, no studies had evaluated the frequency and diagnosis of VCD in asthma patients by applying IOS.

Our data showed the prevalence of 23.53% (12 out of 51 patients) of VCD. This high figure is at variance with the existing literature, which described VCD as a relatively rare differential diagnosis of asthma. This observation may be the first true reflection of the prevalence of VCD among asthmatics. Nine of these 12 (75%) patients with VCD fulfilled the spirometric criteria for reversible airway obstruction. These results support the earlier view that VCD and asthma more often coexist.^1^,^4^ Present data also amplified the observations of previous studies^1^–^5^ that the majority of patients with VCD are young adult females.

There was no significant difference between the VCD-positive and VCD-negative groups in the spirometric parameters. The observations of the PFT parameters of this study agreed with the findings of other studies.^1^,^4^ Only a single case in the VCD-positive group had inspiratory limb flattening of the FV loop, which had been considered characteristic of this entity. Previous studies have indicated low utility of the FV loop in conclusively diagnosing VCD, as the sensitivity of the FV loop observed in even known cases of VCD was very low. In two separate studies, the characteristic flattening of inspiratory limb of FV loop was reported in only 23%^1^ and 20%^4^ of proven VCD patients.

In our study, the upper airway influence was evident on IOS in seven (58.3%) of patients in the VCD-positive group and in six (15.4%) of patients in the VCD-negative group. This difference was statistically very significant (*P* < 0.005). We took the appearance of the oropharyngeal compliance *C*_m_ on visual interpretation graph as evidence of upper airway influence. The major problem of the FOT is that its results are influenced by the shunt characteristics of the upper airway. Part of the oscillations applied at the level of mouth is ‘lost’ in movements of the cheeks and floor of mouth. This shunt does not influence markedly the measurements in healthy individuals, but will play a progressively larger role when the impedance of the respiratory system is abnormally large.^10^ The same shunt influences the values of *R*_rs_ and *X*_rs_ in patients of upper airway obstruction and COPD, but in latter case, a shunt is also operating at the level of intrathoracic central airways.^11^,^12^

Hence, oral shunt is the only shunt operational in upper airway obstruction. In the Mead's model of lung [Figure 1], this oral shunt is summarized under the term oropharyngeal compliance, *C*_m_. VCD is a cause of upper airway obstruction and oral shunt plays a role in such patients.

IOS has not been used in the evaluation of VCD until now. Our results indicate that IOS can be a useful screening tool for suspected VCD cases. In addition, IOS offers certain advantages over spirometry as it is effort independent, consumes less time, and requires minimal patient cooperation.

In conclusion, VCD should be considered as a common differential diagnosis in young adult females who are being treated for asthma, especially if the symptoms respond suboptimally to optimal therapy. Spirometry in patients of VCD usually demonstrates reversible airway obstruction due to the underlying asthma, which often coexists with VCD. FV loop infrequently demonstrates the characteristic inspiratory limb flattening. IOS is a useful screening tool for the presence of VCD in patients of asthma. The definitive diagnosis, however, depends on direct visualization of the paradoxical vocal cord adduction on inspiration either during quiet respiration or with provocative maneuvers.

## References

[1] Newman KB, Mason 3rd UG, Schamling KB (1995). Clinical features of vocal cord dysfunction. Am J Respir Crit Care Med.

[2] Rodenstein DO, Fancis C, Stanescu DC (1983). Emotional laryngeal wheezing: A new syndrome. Am Rev Respir Dis.

[3] Ramirez RJ, Leon I, Rivera LM (1986). Episodic laryngeal dyskinesia: clinical and psychiatric characterization. Chest.

[4] Morris MJ, Deal LE, Bean DR, Grbach VX, Morgan JA (1999). Vocal cord dysfunction in patients with exertional dyspnea. Chest.

[5] O'Connell MA, Sklarew PR, Goodman DL (1995). Spectrum of presentation of paradoxical vocal cord motion in ambulatory patients. Ann Allergy Asthma Immunol.

[6] Beale HD, Fowler WS, Comroe JH (1952). Pulmonary function studies in 20 asthmatic patients in the symptom free interval. J Allergy.

[7] Christopher KL, Wood 2nd RP, Eckert RC, Blager FB, Raney RA, Souhrada JF (1983). Vocal cord dysfunction presenting as asthma. N Engl J Med.

[8] McFadden ER, Zawadski DK (1996). Vocal cord dysfunction masquerading as exercise induced asthma: A physiologic cause for “choking” during athletic activities. Am J Respir Crit Care Med.

[9] Muller E, Vogel J (1981). Modeling and parameter estimation of the respiratory system using oscillatory impedance curves. Bull Eur Physiopath Respir.

[10] Ritz T, Dahme B, Dubois AB, Folgering H, Fritz GK, Harver A (2002). Guidelines for mechanical lung function measurements in psychophysiology. Psychophysiology.

[11] Van Noord JA, Wellens W, Clarysse I, Cauberghs M, Van de Woestijne KP, Demedts M (1987). Total respiratory resistance and reactance in patients with upper airway obstruction. Chest.

[12] Vogel J, Smidt U, Vogel J, Smidt U (1994). Impulse Oscillometry.

